# The consultation of rugby players in co-developing a player health study: feasibility and consequences of sports participants as research partners

**DOI:** 10.1186/s40900-017-0055-1

**Published:** 2017-05-04

**Authors:** Madeleine A.M. Davies, Edward Balai, Jo Adams, John-Henry Carter, Andrew Judge, Julia L. Newton, Nigel K. Arden

**Affiliations:** 1 Oxford NIHR Musculoskeletal Biomedical Research Unit, University of Oxford, Oxford, UK; 2 Arthritis Research UK Centre for Sport, Exercise and Osteoarthritis, University of Oxford, Oxford, UK; 3 Medical Sciences Division, University of Oxford, Oxford, UK; 4 Faculty of Health Sciences, University of Southampton, Southampton, UK; 5 Oxford University Rugby Football Club, Oxford, UK; 6 MRC Lifecourse Epidemiology Unit, University of Southampton, Southampton, UK

**Keywords:** Player, Involvement, User, Design, Public, Health, Consultation, Rugby

## Abstract

**Plain English summary:**

Many funding bodies within the United Kingdom and globally have encouraged public involvement in research. The Department of Health has also called public involvement a sign of good research. Despite the wide acceptance of public involvement improving many aspects of research, from its design to its communication, involvement has varied levels of implementation across different fields of research. Sports people have rarely been involved in research, partly as this research tends not to be funded by mainstream funding bodies. This may lead to a lower research quality, not founded in player (‘service user’) experiences.

When creating a study of former rugby player health, we were very keen to involve rugby players, understand their thoughts on player health, and their playing experiences. This article explains how rugby players were involved in several ways, but mainly in group discussions during the design stage. These groups helped to inform our study’s aims and questionnaire, ensure the questionnaire would capture player experiences and answer questions relevant to players, that they would like to understand after their participation in rugby.

We found that these groups were easy to arrange, and that in only one session with each group, we were given many ideas of how to improve the questionnaire and study. We believe that other studies in sports should involve sports people, and that this is a useful activity that will change data collection forms and processes, improving the research, helping researchers, and making studies more suitable for players who take part in them.

**Abstract:**

**Background** Patient and public involvement (‘involvement’) in the UK has increased in accordance with funding requirements, patient-centered health policy initiatives and reporting of the positive impact of involvement for those involved, research and researchers. However, involvement has not been implemented equally across all disease areas and populations. The aim of this process was to involve rugby players across the research cycle of a player health study, ensure the study is player-centred, and that players had approved and informed the design of the study and its questionnaire from their playing experiences.

**Methods** Two group discussions were undertaken with current students who were playing rugby at a Collegiate University. All male and female University rugby players and two College rugby teams were approached to become involved. Sessions were chaired by a player-lead using a topic guide and were audio-recorded and transcribed. Player suggestions were extracted by the player-lead and discussed within the study team for inclusion in the player health study and its questionnaire.

**Results** Players readily engaged with the sessions and made many contributions to the development of the study and the questionnaire. Players discussed whether certain topics were being collected satisfactorily, and whether the questionnaire would encompass their playing experiences or that of other players. Players suggested where answers might be less reliable, and ways in which this could be improved. Players recommended additions to the questionnaire, and questioned researchers on the choice of language, motivation for question inclusion and if measures were standardised or novel. Alterations were made to the questionnaire based on suggestions, where these were agreed by the study team.

**Conclusions** Involving a group of players in the design of a player health study and questionnaire was not an arduous process and was rewarding for researchers. The process resulted in numerous alterations to the questionnaire and its functionality, which may improve response rate, the experience of players participating in the player health study, and their ability to report relevant information aligned with their previous experience. Player involvement in research was feasible to implement and improved not only the questionnaire, but also researcher confidence in the project and player experiences being accurately captured and leading a reliable data collection processes in a population with the potential for cultural bias to affect the ascertainment of health, pain and injury.

## Background

Public involvement (‘involvement’) is defined as “research being carried out ‘with’ or ‘by’ members of the public, rather than ‘to’, ‘about’ or ‘for’ them” [1] and is now widely seen as a component of good research practice, increasing its quality, relevance and accountability [2–4]. Involvement has also recently become a prerequisite for NIHR funding [5] and national guidelines to assist with the integration of involvement into health service research have been developed by advisory group INVOLVE [1].

Health policy has been central to the growth of involvement in research across several developed countries, with the National Health Service Research and Development Strategies calling for its integration into the healthcare system and healthcare research [6]. Involvement is encouraged by the Department of Health, who state that ‘relevant service users and carers or their representative groups should be involved wherever possible in the design, conduct, analysis and reporting of research’ [7]. The term ‘Public and Patient Involvement and Engagement’ includes members of the public, as citizens with the potential for health service utilisation, and also service users who are current patients or their relatives and carers, who may have a specific investment in improving services and research, and ensuring their views and experiences are included in decision-making within healthcare.

The need to strengthen reporting and measurement of impact within involvement has been previously discussed [4, 8]. Tokenistic involvement, whereby the mandatory inclusion of involvement on funding applications, and as a sign of good practice, may contribute to involvement being considered a ‘tick box exercise’, has been previously considered [4, 9]. Strengthening the reporting of involvement and encouraging publication has been considered as one method of preventing conscious or subconscious tokenistic involvement within research [4]. The importance of working with a group that will provide the knowledge and perspective that research teams would like from members of the public and may be missing from their team has also been discussed as central to successful involvement [1, 8].

Involvement can be appropriate for all stages of the research process [5] including research bids [10], naming and branding studies [11], developing interventions [12, 13], trials [2] or placebo design [14]. Despite the recommendation of involvement across all stages of research and for all relevant patients or service users, involvement has not been implemented equally across different disease areas and populations with an International bibliometric review of involvement in health research demonstrating that involvement has traditionally being undertaken more so with black and minority ethic and indigenous populations, and most prominently research areas of mental health, indigenous population health and cancer, followed by sexual health, children and parenting, and diet, obesity and diabetes [15]. This clustering amongst populations and illness areas may be as these groups are more regularly being involved in healthcare and research, the chronic nature of these conditions permitting the rapport and the opportunity to undertake involvement activities or that these endeavours are more regularly documented [15] or undertaking progressive and impactful involvement.

The public has been described by INVOLVE as including ‘patients, potential patients, carers and people who use health and social care services as well as people from organisations that represent people who use services’ [1]. In research that is not focusing on healthcare services or patients, the importance of involving those specific populations and understanding their experiential knowledge may be equally valuable to the research process, those involved, and researchers.

Despite the growth of involvement and its widespread adoption across many different disease areas and research stages [15], there has been limited application of involvement to studies involving sporting populations, either to involve them as citizens, or a specific population with the potential for increased service use following sports participation. With the benefits of involvement for researchers, members of the public and research quality now well-documented [1, 8, 16, 17], there is a need to emphasise the ease of integrating involvement into studies involving sports participants, and to encourage these fields to adopt the good practice of other areas of healthcare science and medicine. This will not only increase research relevance, quality and potentially recruitment, by providing accessible patient-facing documents, but also give voice to those who will ultimately be affected by research outcomes, as service users with a specific identity that may affect treatment decisions and their perception of clinical processes and judgments.

The motivation for involvement in research has previously been described as epistemological, moralistic and consequentialist [18]. Epistemological motivation for involvement is the experiential knowledge that can be bought to the project, its processes and researchers by involving specialists in that environment, such as patients, carers and service-users. Moralistic motivation has been described as the involvement of the public in research for moral, ethical or public right [18]. Research that is publicly-funded or charity-funded should be involving those who are funding this work in order to improve transparency and that those likely to be ultimately affected by the outcomes of research, should be contributing to this research. The consequentialist or effectiveness argument describes how research can be enriched with the involvement of members of the public and that there can be improvements in its quality, relevance and impact with involvement [19].

### Experiential knowledge in rugby union

The experiential knowledge of sports participants is extensive and unique to their specific sport’s environment. Sport-specific terminology may represent a unique dialect and shared values of team members have been previously discussed as contributing to a sport-specific subculture or individual society [20]. The experiential knowledge of players about their own environment, sport’s ethos and attitude to health and research are unique, and involving players in research is the only way to ensure player experiences are truly encompassed and represented within a research study.

Involvement may be particularly beneficial in areas where groups may be hostile to services or service providers [8]. Due to recent widespread scrutiny of player health in rugby union [21–23], understanding players’ attitudes to research and researchers will assist with anticipating player response and mitigating players concerns surrounding the portrayal of a sport, where player values may result in an unwillingness to cast the sport in a negative light [20].

In addition to increasing the quality and relevance of research and meeting demands of public accountability and transparency, the unique sporting subculture of rugby union has been previously acknowledged as possessing a cultural non-acceptance of pain and injury [24, 25]. Involving players as co-researchers, collaborators and consultants throughout various stages of this research project was hoped to increase the accuracy of data collection and self-reported outcomes of pain and disability, within a population historically appreciated to consciously disregard pain and injury [25].

Whilst not previously having been documented as involved in collaborative research, there is precedent for involving the most relevant members of the public with a specific project. It has been recommended to consider the knowledge and perspective that you are looking for from members of the public when working with them with a research project [1]. Involvement has been effectual in scenarios where specific populations have higher levels of health inequality, but also may have contrasting worldviews or cultural values that either contribute to this, or impede progress to reduce inequalities [26, 27].

### Can sports people be considered marginalised in healthcare?

Research within sports science exploring physiology, psychology, nutrition or increasing elite performance and health, or research within sports medicine exploring injury, pain and pathology, have thus far been extremely limited in their involvement of members of the public, or sports participants.

Sports participants may not traditionally be viewed as disempowered or marginalised, however could be viewed as such when considering their capacity to make individual and unconstrained choices with regard to their health and healthcare, such as surgery or return to play decisions. Within elite sports environments, numerous factors such as squad availability, league placement, upcoming fixtures and player motivation may influence healthcare decisions. The healthcare decision-making process may encompass a team of support staff with varied interests, including coaches and managers, institutional healthcare providers such as team physicians, medical officers or national institutes for sports. The complexities of return to play decisions in elite sports, such as low-compliance with return-to-play procedures, and whether a full recovery from injury is needed in order to train or compete have been previously considered, with 12% of club personnel within New Zealand rugby clubs believing a full recovery was essential for a return to competition [28–30].

Amateur or recreational players may not experience the same pressures as professional athletes in terms of managing injury in the professional sporting environment, but this specialist environment also often provides the opportunity for novel and comprehensive injury treatment and management. Recreational athletes who are otherwise healthy members of the public requiring generally acute primary care utilisation for a specific injury may not be treated by clinicians with expertise in Sport and Exercise Medicine, who are aware of their potential environmental pressures or sporting demands. In comparison to other service users, recreational athletes may also be not as actively involved in patient-centred care or ensuring their needs are adequately met, possibly as a result of shorter admissions, preventing athletes becoming the traditionally more involved chronic morbidity populations [15]. In addition to limited evidence of recreational athlete involvement in health service provision, there has also been little historical involvement as co-researchers or collaborators within research studies.

This article documents the different forms of involvement of current and former rugby players, as a specific group of service users, across various stages of the research cycle in an English study of former rugby player health and the feasibility and benefits of integrating player involvement into sporting research practices. A primary consideration in the methodology of this player health study was significant player involvement and research being player-centered.

Involvement during the study took place in several ways:

1. Collaborative study management roles
2. Individual scoping discussions with current and former, recreational and elite players during the design phase and before the dissemination of results
3. Consultancy in ‘fora’ (plural of forums) informing study design, questionnaire content and recruitment methodology
4. Collaborators in testing data collection platforms for ease of use and test-retest validity reliability

This article will focus particularly on the fora: the format, suggestions and outcomes of this, and ways in which involvement may be better implemented in sporting populations in the future. The authors are aware of discussion surrounding the classification as members of public as ‘patients’, ‘participants’, ‘consumers’, ‘collaborators’, ‘co-researchers’ and ‘consultants’ depending on the research area and level of involvement. Within this article members of the fora involved in the design stages of the project will be referred to as ‘players’ as they were approached due to their experiential knowledge as a result of this status.

The aim of the fora were to involve current players in the design and development of an accessible and player-focused questionnaire, with limited bias resulting from the sport’s sub-culture and values.

## Method

The player health study was a cross-sectional questionnaire study with retrospective data collection, the results of which will be reported elsewhere.

The setting of the fora was a high-ranking Collegiate University. The entirety of the University’s male and female rugby teams, and two specific College rugby teams were approached through mailing list administrators to attend one of two sessions being coordinated at the aforementioned Colleges. Details of the sessions’ aims and the player health study were included in this initial approach email. Session times were scheduled according to player availability, and once timings were agreed these sessions were again publicised using social media and on the University’s rugby mailing lists.

Players who expressed an interest following the initial call and confirmed their availability for a session were sent further details of the session’s structure. They were informed of the player health study’s ethical approval and that the session would be an informal open forum discussion of their thoughts and views on the structure and content of paper and online-format questionnaires to inform the project’s development.

Sessions were audio recorded and transcribed by the player-lead (EB), and then reviewed by a member of the study team also present at the sessions (MD). All data collection and processes followed good research governance principles.

The fora were not intended to be the research activity of qualitative focus groups seeking to generate new knowledge, but were designed to be player-led group discussions where players could inform researchers in designing and implementing an appropriate questionnaire and study design, which would accurately encompass player experiences and capture elements of player health that players felt needed to be better understood.

### The fora

Two fora were carried out with amateur level, University rugby players. The player-lead was a current player and medical student on a research placement with limited prior exposure to the research project. Eleven players participated in the fora, with five in one session and six in the other. Ten were male and one was female.

The multidisciplinary research team produced a topic guide for the sessions with the fora lead in collaboration with an experienced qualitative researcher. Players involved in the fora did not receive any research training prior to the fora, but the player-lead had several meetings with the research team, was given an overview of the project and attended two meetings discussing the topic guide and conducting fora lead by a qualitative researcher experienced in chairing focus groups.

Players were informed that information disclosed to the group would be anonymous and treated confidentially, and that the aim of the session was questionnaire and study feedback and that there were no right or wrong responses. Further prompts and probes were used in conversation when appropriate.

Key suggestions were extracted from transcripts by the player-lead and a researcher present at the sessions. Player suggestions were discussed by the research team (JN, NKA and MD) and implemented where possible by a researcher who had been present at the fora (MD). Suggestions mentioned in both fora, or repeatedly by different players within a group were prioritised for inclusion.

## Results

Both fora communicated easily, freely discussing their playing experiences in order to inform the design of the study. During the review of the paper questionnaire, some players ask questions which were often answered or considered by other members of the group, before clarification was sought from the player-lead.

Players openly justified their comments and opinions with their previous playing history experiences. Group consensus was not sought or encouraged, and the groups appeared comfortable to disagree and demonstrate contrasting views. However players mostly agreed on concepts and considered issues in an iterative manner, demonstrating similar experiences and contributing to each other’s ideas.

Specific consequences from the fora and ensuing key questionnaire or process alterations are outlined in Table 1.

**Table 1:** Resulting alterations from the fora

Player’s Suggestion	Change resulting from player involvement
Use event markers	Event markers created for some questions, such as ‘How often were you concussed?’ becoming ‘How often were you removed from play due to concussion?’ to minimize recall bias.
Use ratios for incidence	Categories of ‘every (n) games’ to assess the incidence of more frequent injuries.
Long-term outcomes associated with other sports or injuries being separate from rugby	Categories of training, playing and non-rugby created for each injury reported.
Save and come back later	Players are now able to save information and re-enter the platform at a later point in time, to assist with sections such as Family History where this information may not be immediately available.
Reorder in order of importance	Current pains and primary outcome sections were placed earlier in the questionnaire.
Alter occupation categories	An additional category was added so players can put ‘professional sports player’ which previously would have had to be ‘skilled manual worker’.
Include mental health status	Questions on anxiety and depression were added as players felt that this was relevant and that it would not act as a deterrent for participation

### The groups’ views

Players were keen to ensure that individual questions were phrased optimally and achieved this by relating their experience and understanding of the question back to researchers and each other, such as: ‘When you’re asking about keyhole surgery or cartilage, have you got the option of if they’ve had multiple operations?’ Comments such as ‘I would put…’ demonstrated both a consideration of the question’s results, and an immersion in the project that often resulted in suggested alterations to the wording or phrasing. Players suggested where further qualification of questions was necessary and when they would be unable to answer questions: ‘I think ‘What is the average distance you work per week?’ is going to be a tough one to work out’.

Players within both groups voiced concerns about accurately examining the pain of former players. They discussed their own current pains and the differences between acute and more long-term pain, and how pain they experienced differed between their own joints.

Based on players’ experiences and interests, or areas players considered pertinent to this population and important for the game to better understand, questions were suggested as additions to the questionnaire. Players were also directly asked what they would add to the questionnaire, and suggested the availability of medical staff and support following an injury to be important to understand. The way that injuries affected your ability to work was considered both a valid marker of severity and also important to players in terms of its impact on their daily life.

There were many questions from players to researchers during the sessions. This was mostly surrounding the reasoning behind specific question choices, whether questions were standardised ways of collecting concepts or what specific information was required from a question, so players could consider if the concept was being satisfactorily captured with that question. This made researchers accountable to the group, which was an initial aim in involving players in the project.

Players were made aware in the introduction to the session that the questionnaire would be sent to former players at various amateur and professional levels. The proposed recruitment strategy for the study was discussed with players, who were able to inform researchers of potential motivators which would encourage their participation or had encouraged them to participate in previous rugby initiatives, and factors which may dissuade questionnaire completion, such as questionnaire length, boredom and extensive drop-down lists. Players discussed the differences between former and current players and often referred to the possible contrasts in answers, player experience and outlook between current and former, and amateur and professional rugby players. Within this context, some players referred to their ‘dad’s generation’ as the older, target audience. They discussed recall bias, changes within the sport over time and a contrasting mentality between older and younger players.

Players regularly discussed their own experiences, and also those of other individuals in games they had played in, or elite level games they had watched. This confirms the epistemological motivation for player involvement and enables question alterations to mirror players’ experiences or circumstances they had witnessed other players experiencing which would not have been accurately captured in the initial questionnaire.

## Discussion

### Sports participants as research partners

Current players were involved in this process as expert consultants to inform the study design, questionnaire and research agenda. This is the first occasion we are aware of sports participants being involved in informing research with their experiences. Within these sessions, players assumed the role of researchers, discussing how certain questions could be phrased to be most accurately answered, and examining the bias that may be introduced with certain terminology. Female and male clubs were approached to become involved in the fora. The uptake of female players was notably less than for males, however this may be a reflection of participation in rugby union throughout the University, with more male players involved at a College and University level providing a greater number of male attendees.

Players often asked researchers if they had considered certain elements, issues or topics. This contributed to addressing content validity in the questionnaire and also held researchers accountable to the fora, not only ensuring the effectiveness of questions and their outcomes, but also demonstrating involvement as a form of empowerment [18], for a group who may potentially be affected by the outcome of a study to have been involved in its objectives, priorities and design.

Within this consultant role, topics of questionnaire fatigue and recall bias were raised, considered and addressed by players. This resulted in researchers observing a similar process to those experienced during initial questionnaire design, and appeared to validate some of the reflection processes researchers had previously navigated. The concept of players exploring and vocalising similar considerations to researchers may be due to similar experiences between players and the research team, or similarities in the values, assumptions and priorities of players and the researchers. The impact of involvement has previously been considered to be greatest where the skills and experiences of those involved differ from the research team [8]. As researchers and players were both undergraduate and postgraduate students at the same University this similar reflection process may be a result of shared values and experience.

The pursuit of a democratic approach to healthcare provision and research, which has driven the growth of involvement in research, has been discussed as empowering healthcare users and patient representatives to contribute to the research process and hold researchers accountable in publicly funded research. Methods of actively empowering patients and members of the public have previously been suggested [31, 32], and research training for the public has been discussed as enabling members of the public to critique the research cycle more effectively and improve public confidence in articulating their sentiments during involvement [17].

During these fora, possibly due to the specific sporting population, players’ prior acquaintance with each other at a University or College level, or their status as students of a high-ranking University, players easily assumed a critiquing researcher role desired within public consultations. Players were also comfortable to appraise the questionnaire and research team for prior decisions, without a specific focus of the session being their empowerment or a notion of perceived power exchange between researchers and players being stressed. Players did not receive specific research training, which has been recommended for involvement, and described as assisting with more effective critique of research by those involved [1, 17]. However, as student athletes with multiple demands on their time, researchers were concerned that an additional training session would have reduced attendance and proved too demanding for those involved. The tutorial system in which many of the student-athletes will participate during their studies may also influence their ease in discussion, as this group may be acclimatised to voicing opinions, contributing in a discussion forum and developing each other’s ideas.

Training in involvement also introduces concerns of professionalization, with the potential of losing a ‘lay perspective’, either directly through training activities, or as a result of research knowledge and experiences gained through multiple projects [33, 34]. Researchers were concerned that players undergoing training in research methodology may lose an ideological lay perspective, which was of substantial value to researchers, more so than obtaining critique on an even footing, which may have been less applicable for this player population.

The involvement of a rugby-playing medical student as group lead who was previously acquainted with the majority of players, and had experienced limited prior exposure to the study with no official study role, but an awareness of the project and session aims, may have facilitated the groups’ interaction and success. The notion that user involvement is facilitated by a perceived lack of significant operationalized power structure between the users and researchers, and that the perception of equality is vital has been previously discussed [10] and may have facilitated dialogue during these sessions.

Consultative fora in the design phase are only one method of involvement within this player health study. Current and former amateur players were involved in initial study design discussions and there are player representatives on the Study Steering Group, directly informing study processes and being involved with interpretation of study results and publication strategy decisions, in addition to future study direction.

### Session Structure

A topic guide was used to broadly structure conversation and receive focused feedback on specific perceived weaknesses within the questionnaire and study design. The topic guide followed the questionnaire structure, from first impressions through to any information participants thought was omitted and should be included. This allowed players many opportunities to comment on specific questions, ensured the entire questionnaire was covered and allowed high level and specific feedback, with some inter-group consensus as players moved through the questionnaire together, in a relatively short session, which were both between one and two hours.

The session format of two fora, led by a student with a topic guide sufficiently detailed to stimulate conversation, address areas of researcher concern and provide session structure, without relying on researcher input, allowed the session to be player-led. The research team was mindful of allowing the groups to feel capable of expressing opinions and concerns without having to, or feeling that they must find faults or make alterations to the study.

A member of the study team (MD) was present as an observer for the sessions. This provided the opportunity to observe the initial reaction of players to the questionnaire. Interpretation of these responses provided insight for the quantitative study team into potential motivators and deterrents for participation upon receiving the questionnaire, which has influenced methods of recruitment by including incentives, encouraging clubs to be ambassadors for the research, involving the teams medical staff, considering timing in the season when questionnaires are sent, and considering designated times when players can participate together.

### Strengths and weaknesses

The involvement of players within this questionnaire and study design was an informative process for researchers, resulting in several changes to the questionnaire (Table 1) and increased confidence of researchers that the questionnaire was approved by players and would collect player-centred information in the most precise way.

The questionnaire structure, specific questions and online functionality were altered and improved following recommendations. Questions that may be more difficult for players to answer were highlighted to researchers during the project, even if the fora or study team felt limited in scope to alter this whilst maintaining the study objectives.

As a consultative activity, the sessions were player-lead by one particular player, who had delegated responsibilities for the sessions from the study team, as opposed to being truly lead by a group of player-partners in collaborative decision-making that would be directly implemented. Researchers felt that player-collaboration was present in other aspects of the study, and that the purpose of this session was more targeted feedback, which lead to a consultative approach using a topic guide for structure.

The groups for this exercise were locally available to researchers and currently playing at an amateur level of play. A player involvement session within an older group may have been useful in order to receive any additional feedback from a group with different experiences, who had also played in the amateur era of the sport and may have a different approach to the questionnaire based on these experiences. Fora attendees being current amateur players may however benefit the study by ensuring the relevance of the research project to this population who will be influenced by the translated results, and who also form the majority of the playing population.

Further player involvement in the application of alterations and suggestions may have helped to ensure suggestions were embedded without bias from the study team, and that players maintained their authority throughout the process. However, researchers were concerned with burdening players, and the team felt that as ambassadors of involvement, player suggestions would be integrated where possible. The main reasons for non-inclusion were where player suggestions may be less relevant for former players or where a suggestion would involve multiple smaller questions to be accurately captured, therefore taking too much space within the questionnaire for a secondary study objective.

The need for more critical evaluation of involvement activities has been previously discussed and efforts have been made to improve the reporting of such activities and evaluate them [35], to prevent involvement being treated as a tokenistic exercise [4]. The number of changes in the questionnaire as a result of the fora, and process of players considering research questions, limitations and outcomes using their previous experience to inform this demonstrates the worth of these sessions within this project. Players did not, however, formally evaluate the process as has been suggested previously in the literature [14, 35], although players also did not request to do so.

### Key reflections on process

#### Benefits

- □ Current sports players willingly gave their time without incentives or any payment, and readily discussed the questionnaire, altering the way key variables were collected.
- □ Novel thoughts were presented to researchers at a stage where they could be incorporated into the study.
- □ Researchers developed increased confidence in the questionnaire’s appropriateness and acceptability for players.

#### Difficulties

- □ Specific funding was not available for this activity and whilst the groups were coordinated at locations convenient for players meaning researchers travelled to the groups rather than players, payment for patient involvement has been encouraged [1, 14, 17].
- □ Training may have enabled more effective critique and improved confidence for players.
- □ Older players may have had alternative views that were not encompassed in these fora.
- □ The number of players taking part in the fora was notably lower than those approached to participate, particularly for female players. However, this process was developed to assess the feasibility of players being involved and consult players on improving the questionnaire and study processes, and not to qualitatively examine generalisable players’ views.

### This process in the context of previous involvement work

It has previously been suggested that involvement is limited to personal experiences by a lack of research training [16]. However, researchers within this project found increased value in this, as players bought a wealth of personal experiences and idealistic views that were not restrained by methodological or logistical considerations. These fora were coordinated with limited resource and a desire to cause minimal player inconvenience, and therefore scheduling a training element to the session may have decreased adherence and altered the demographic of those choosing to attend.

As an involvement process, these fora were not intended to answer a research question. The sessions aimed at understanding how to develop and design our study and questionnaire to be accessible and player-focused. We also sought to gain insight into some of the identified issues relating to the reporting of pain in sports players and any potential modifiable barriers to acceptance and completion within this sporting cohort. This article has summarized conversation and key resultant action points within these fora and confirmed involvement to be a research-altering process within this population.

## Conclusions

This process has demonstrated the relative ease of establishing a group of amateur sports players, as a form of involvement, and the benefits of involving sports participants in athlete-focused research studies. This exercise has demonstrated that player involvement can support the foundations, goal setting and outcomes of research projects, and potentially improve the collection of sport-specific variables, as informed by player experiences.

We believe this is the first time that rugby players have been involved as expert consultants to help design, develop and review sports research in the initial stages of research development. We anticipate that this process will strengthen the recruitment of our study and ensure that the self-report questionnaire developed is relevant, player-centred and subject to minimal population-driven bias.
